# Dietary Habits and Nutrient Deficiencies in a Cohort of European Crohn’s Disease Adult Patients

**DOI:** 10.3390/ijms24021494

**Published:** 2023-01-12

**Authors:** Fernando Rizzello, Paolo Gionchetti, Enzo Spisni, Ilaria Maria Saracino, Irene Bellocchio, Renato Spigarelli, Noemi Collini, Veronica Imbesi, Thierry Dervieux, Patrizia Alvisi, Maria Chiara Valerii

**Affiliations:** 1 IBD Unit, IRCCS, Azienda Ospedaliero-Universitaria di Bologna, University of Bologna, Via Massarenti 9, 40138 Bologna, Italy; 2 Department of Biological, Geological and Environmental Sciences, University of Bologna, Via Selmi 3, 40126 Bologna, Italy; 3 Microbiology Unit, Department of Specialized, Experimental and Diagnostic Medicine, St. Orsola Hospital, University of Bologna, Via Massarenti 9, 40138 Bologna, Italy; 4 Prometheus Laboratories, 9410 Carroll Park Dr., San Diego, CA 92121, USA; 5 Pediatric Gastroenterology Unit, Maggiore Hospital, Largo Nigrisoli, 2, 40133 Bologna, Italy

**Keywords:** Crohn’s disease, vitamins, mineral, nutrient deficiency, intestinal malabsorption

## Abstract

Wrong dietary habits, such as the Western-style diet, are considered important risk factors for the development of Inflammatory Bowel Diseases (IBDs). Nevertheless, the role of dietary patterns in the clinical management of IBD patients has not been fully investigated yet. Fifty-four patients diagnosed with active Crohn’s disease (CD) were enrolled and subjected to nutritional intake analysis through a weekly food diary. Nutritional patterns were analyzed, and nutrient intake was compared with those of 30 healthy subjects (HS). Blood levels of cholesterol, folic acid, minerals (K, Mg, Fe) and amino acids, were measured in CD patients to assess the presence of nutritional deficiencies. CD patients, with respect to HS, consumed significantly lower amounts of fiber, vitamins (A, E, C, B6, folic acid) and β-carotene. Their calcium, potassium, phosphorus, iron, magnesium, copper and iodine intake were also found to be significantly lower. In blood, CD patients had significantly lower concentrations of total cholesterol, potassium, iron, and amino acids. Active CD patient diet was significantly different from those of HS and may contribute to the establishment of nutritional deficiencies. Intestinal malabsorption was evidenced in these patients. Correction of the diet with specific nutritional plans is a necessary therapeutic step for these patients. ClinicalTrials.gov: NCT02580864.

## 1. Introduction

Inflammatory bowel diseases (IBDs) comprise Crohn’s disease (CD) and ulcerative colitis (UC), two chronic, inflammatory disorders of the digestive tract that develop in adolescence and early adulthood and overall affect about 1.5 million Americans and 2.2 million Europeans [1]. IBDs are characterized by an uncontrolled immune-mediated inflammatory response in genetically predisposed individuals exposed to environmental factors, collectively referred to as the exposome. The exposome concept includes diet, medications, nicotine, infectious agents, stress and lifestyle which contribute to the trigger of the gut chronic inflammatory loop [2]. The shift of the population to Western-style dietary habits, called the Western diet, is considered an important risk factor for IBDs [3]. This hypothesis is sustained by epidemiologic studies showing a rise in incidence and prevalence in people emigrating from low-prevalence to high-prevalence countries and an even more evident increase in their children [4]. The Western diet, characterized by the consumption of high amounts of animal proteins, saturated fats, and processed foods and by low amounts of vegetables, unsaturated fats, fibers, and fruits, may trigger a proinflammatory environment in the gut in susceptible individuals through an alteration of the gut microbiome and an impairment of the epithelial barrier functions [5,6]. Proper nutrition is important in prevention but also in the management of patients with active IBD since they are at an increased risk of nutrient deficiencies. Malnutrition can occur both in UC and in CD, and its prevalence in IBD patients ranges between 20% and 85% [7]. Weight loss occurs in 70–80% of hospitalized IBD patients and in 20–40% of outpatients with CD [8,9]. The prevalence of proteins, energy or specific nutrient unbalance is higher in CD compared to UC, because CD also affects the small bowel, where nutrient absorption occurs [10]. Malnutrition is defined by the World Health Organization (WHO) as deficiency, excess, or imbalance in a person’s intake of energy and/or nutrients [11]. The European Society for Clinical Nutrition and Metabolism defines malnutrition as a state resulting from a lack of intake or uptake of nutrients that leads to altered body composition (decreased fat free mass and body cell mass) leading to diminished physical and mental function and impaired clinical outcome from diseases [12]. Most of the studies focusing on nutrition in IBD have addressed the effects of particular diets in helping the induction of remission of the disease and in contrasting macro- and micronutrient deficiency evidenced in the blood of these patients [13,14,15]. This study aims to analyze the eating habits of patients with active CD, in the absence of nutritional guidelines, in comparison with those of an age-matched healthy subjects’ group (HS) to understand if and how the presence of an active disease with gastrointestinal symptoms can influence eating habits. This analysis has been extended to the presence of nutrient blood deficiencies to understand if malnutrition could be linked to intestinal malabsorption or rather to a reduced nutrient intake. This study was performed in the context of a one-year prospective clinical study aimed at identifying biomarker predictors of the response or failure to standard biologic therapy in CD patients.

## 2. Results

### 2.1. Macronutrient Intake and Dietary Patterns

**Figure 1:**
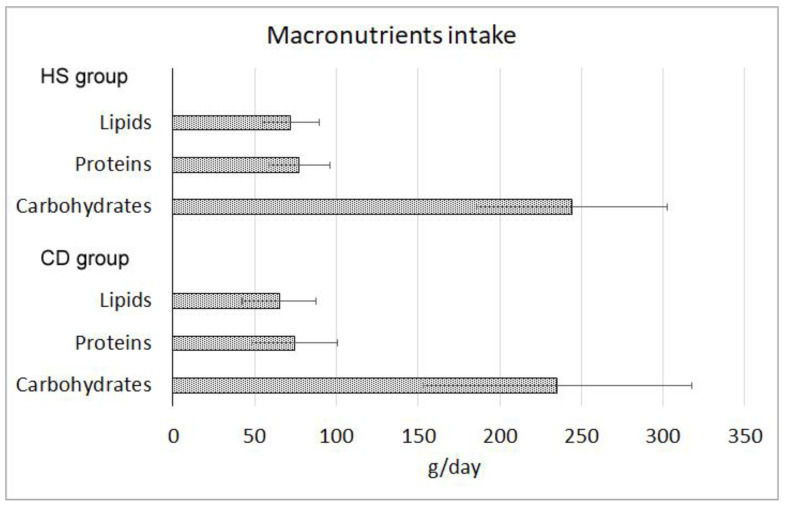
Macronutrient consumption indicated in grams per day (±standard deviation) in healthy subjects (HS) and Crohn’s disease (CD) patients groups. All the differences were not statistically significant (*p* > 0.05).

The total daily caloric intake (measured in Kcal/d) showed no significant differences in the comparison between CD (1824.39 ± 655.04 Kcal/d) and HS (1934.37 ± 468.03 Kcal/d) groups. Furthermore, the distribution of dietary macronutrients (Figure 1) did not show significant differences between groups and fell within the reference ranges of intake suggested for the Italian population by the dietary reference values published by the European Food Safety Authority (EFSA-DRVs). No significant differences were observed in carbohydrates, lipids, or protein consumption between the two groups (Figure 1).

Analyzing the different food groups present in their diet, CD patients showed a significant reduction in vegetable and fiber intake, and these data are confirmed both by soluble and insoluble fibers. Moreover, the CD group revealed a significantly reduced intake of dairy products and fish, but increased consumption of red meat (Table 1). Instead, the intakes of white meat, starchy foods containing gluten and eggs showed no difference between the two cohorts.

### 2.2. Vitamin Intake

**Figure 2:**
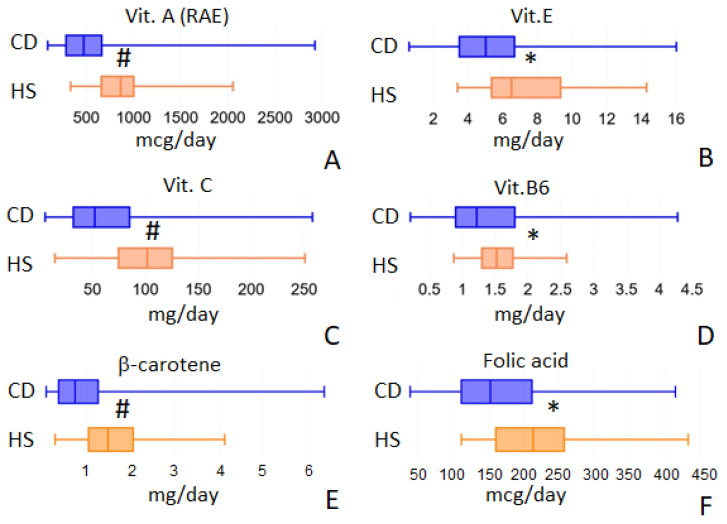
Vitamins intake values that showed differences between the two populations under study: vit. A (panel **A**), vit. E (panel **B**), vit. C (panel **C**), vit. B6 (panel **D**), β-carotene (panel **E**) and folic acid (panel **F**). * = *p* < 0.05; ^#^ = *p* < 0.01.

CD patients were characterized by a reduced intake of vitamins A, E, C, B6 and folic acid (Figure 2A–D). Total vitamin A (RAE, the sum of the retinol and carotenoids activities) decreased from 863.77 (IQR 659.04–1003.57) to 472.02 (IQR 284.69–663.13) mcg/day, *p* = 0.001. Vitamin E, expressed as α-tocopherol, decreased from 0.60 (IQR 0.49–0.89) in HS to 0.46 (IQR 0.31–0.81) mg/day in CD patients (*p* = 0.0451); vitamin C showed a similar trend: from 101.85 (IQR 74.59–125.47) in HS to 52.22 (IQR 31.93–85.09) mg/day in CD, *p* = 0.001. Vitamin B6 decreased from 1.53 (IQR 1.3–1.77) in HS to 1.22 (IQR 0.9–1.8) mg/day in CD patients, *p* = 0.044. Furthermore, folic acid and beta-carotene showed a reduced intake in CD patients. Folic acid intake decreased from 213.42 mcg/day (IQR 160.72–257.5) in HS to 152.94 mcg/day (IQR 111.91–211.89) in CD patients (*p* = 0.002); beta-carotene intake was 1464.78 (IQR 1021.4–2041.36) in HS and 712.14 (IQR 335.53–1240.39) mcg/day in CD patients, *p* = 0.001 (Figure 2E,F).

### 2.3. Mineral Intake

**Figure 3:**
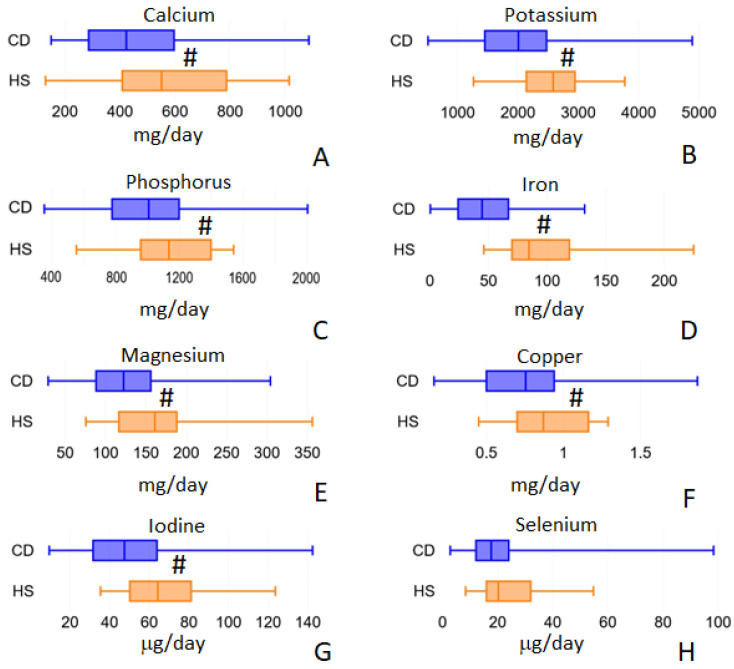
Minerals intake values that showed differences between the two populations under study: calcium (panel **A**), potassium (panel **B**), phosphorus (panel **C**), iron (panel **D**), magnesium (panel **E**), copper (panel **F**), iodine (panel **G**) and selenium (panel **H**). ^#^ = *p* < 0.01.

For mineral intake, we analyzed 15 different elements (calcium, sodium, potassium, phosphorus, iron, zinc, magnesium, copper, selenium, chromium, fluorine, iodine, magnesium, molybdenum and nickel) but we observed significant differences between CD and HS cohorts only for 7 of them: calcium, potassium, phosphorus, iron, magnesium, copper and iodine (Figure 3). Calcium intake decreased from 550.84 (IQR 408.07–787.13) in HS to 422.72 (IQR 286.09–596.5) mg/day in CD, *p* = 0.009. Potassium intake decreased from 2583.63 (IQR 2140.34–2946.35) in HS to 2007.90 (IQR 1451.65–2474.13) mg/day in CD, *p* = 0.001. Phosphorus decreased from 1133.08 (IQR 955.90–1396.84) in HS to 1013.33 mg/day (IQR 775.95–1196.84) in CD, *p* = 0.029. Furthermore, iron intake was found to be decreased from 11.56 (IQR 8.66–12.62) in HS to 8.58 (IQR 6.54–10.69) mg/day in CD, *p* = 0.002. Magnesium intake decreased, from 161.21 (IQR 116.49–188.27) in HS to 122.39 (IQR 88.29–156.12) mg/day in CD, *p* = 0.004. Copper intake decreased from 0.90 (IQR 0.71–1.17) in HS to 0.76 (IQR = 0.50–0.94) mg/day in CD patients, *p* = 0.008. Finally, iodine consumption decreased from 64.34 (IQR 50.22–81.13) in HS to 47.48 μg/day (IQR 31.63–63.89) in CD patients, *p* = 0.001. Selenium only approaches statistical significance (Figure 3H) but fails to achieve it. Its intake decreased from 20.25 (IQR 15.94–32.00) in HS to 17.66 (IQR 12.14–24.02) μg/day in CD, *p* = 0.091.

### 2.4. Lipids

We found a significantly lower consumption of some saturated and unsaturated fatty acids in CD compared to the HS group, while we do not find differences in total cholesterol intake between cohorts (248.57 mg/day in CD vs. 244.73 mg/day in CD). In detail, lipid analyses are listed in Table 2.

### 2.5. ORAC and PRAL

**Figure 4:**
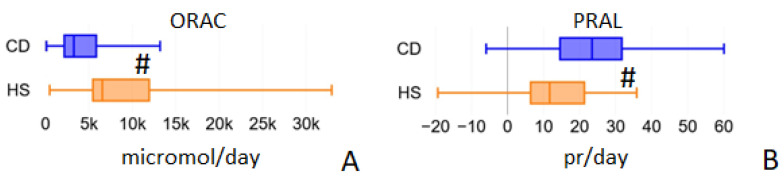
Average daily values of ORAC (Oxygen Radical Absorbance Capacity, panel **A**) and PRAL (Potential Renal Acid Load, panel **B**) calculated by the Metadieta software for the food diaries analyzed for the two populations under study. ^#^ = *p* < 0.01.

**Figure 5:**
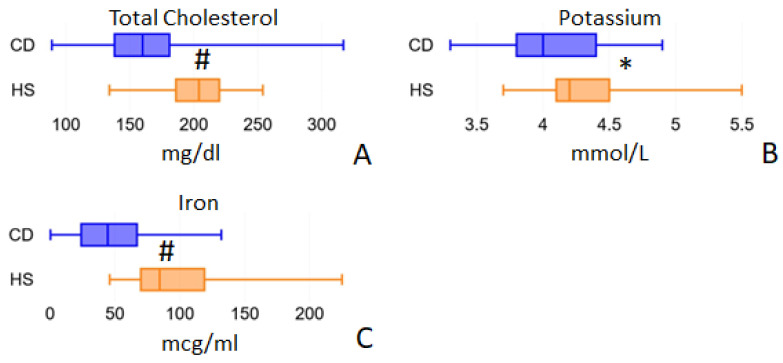
Blood parameters evaluated by routine biochemical analyses that show differences between the two study cohorts: total cholesterol (panel **A**), potassium (panel **B**) and iron (panel **C**). * = *p* < 0.05; ^#^ = *p* < 0.01.

Oxygen Radical Absorbance Capacity (ORAC) of the diet (Figure 4A) was significantly lower in the CD group than in HS: 3257.56 (IQR 2155.64–5837.77) vs. 6530.10 micromol/day (IQR 5446.86–11931.02), *p* < 0.001. This is probably linked to the decreased total polyphenol intake (Figure 5B) observed in CD patients: 290.19 mg/day (IQR 171.37–451.68) vs. 606.22 mg/day (IQR 495.31–880.23) in HS group, *p* < 0.001. On the contrary, the Potential Renal Acid Load (PRAL) of the diet (Figure 4B) was higher in the CD group: 23.43 (IQR 14.55–31.71) vs. 11.70 pr/day (IQR 6.41–21.31), *p* < 0.001.

### 2.6. Nutrient Blood Values

Blood nutrient analysis that we measured included cholesterol, vitamin B12 and B9 (folic acid), minerals (K, Mg, Fe) and amino acids. Magnesium values were almost identical in CD patients at 2.00 mmol/L (IQR = 1.90–2.10) and healthy subjects at 2.00 mmol/L (IQR = 1.90–2.10; *p* = 0.4259). Folic acid blood values were similar in CD patients (5.25 ng/mL (IQR = 3.40–6.90)) and in the HS group (4.75 ng/mL (IQR = 3.70–5.80; *p* = 0.5073)). Furthermore, vitamin B12 in blood showed no significant differences between CD patients (211.50 pg/mL (IQR = 157.00–329.00)) and the HS group (248.50 pg/mL (IQR = 178.00–336.00; *p* = 0.3457)). Total blood cholesterol (Figure 5A) was found to be significantly lower in CD patients than in HS: 160.00 mg/dl (IQR 138.00–181.00) vs. 204.00 mg/dl (IQR 186.00–220.00), *p* < 0.001. However, these reduced values do not depend on a reduced intake, which was completely comparable in the two groups: 248.57 and 244.73 mg/day in CD and HS patients, respectively, *p* = 0.708. Serum Potassium was significantly decreased in CD patients: 4.00 mmol/L (IQR 3.80–4.40) vs. 4.20 mmol/L (IQR 3.50–4.50) in HS, *p* = 0.016 (Figure 5B). Iron was also found to be strongly reduced in CD patient blood: 44.50 mcg/mL (IQR 24.00–67.00) vs. 84.50 mcg/mL (IQR 70.00–119.00) in HS, *p* < 0.001 (Figure 5C). Notably, the median iron blood value recorded in CD patients (43.00 mcg/mL) is close to the minimum physiological limit, and this is in agreement with anemia, which is a frequent condition in active Crohn’s disease patients.

The analysis of the amino acid blood concentrations demonstrated that 12 amino acids (Taurine, Threonine, Serine, Glutamic acid, Alanine, Valine, Cysteine, Methionine, Leucine, Histidine, Arginine, Citrulline) had significantly reduced levels in CD patients, while only Aspartate showed a significantly increased level if compared to HS. It is to be noted instead that their estimated intake was not significantly different between CD and HS groups. The blood levels of each amino acid are shown in Table 3 in comparison with the corresponding estimated intake values.

## 3. Discussion

Our cohort of CD patients has shown to adopt a significantly different diet with respect to age- and BMI-matched healthy control subjects. It is important to underline that these patients showed active disease and received clinical indications to initiate a biological therapy. These symptomatic CD patients tend to consume less fish and dairy products but much more red meat. These changes are balanced from a caloric point of view. The less consumed vegetables (and fibers) did not influence the caloric intake, since vegetables are the components of the diet with the lower energy density. These choices probably represented a voluntary behavior in an attempt to reduce the amount of insoluble fiber which in fact is quantitatively halved in the patients’ food diaries. Dietary fibers are digested by the human microbiome by fermentation reactions within the bowel, important in the maintenance of gut health. However, while it is well established the benefits of the byproducts of fermentation in healthy individuals and their potential health benefits in other diseases, it remains to understand how fiber may affect gut health in dysbiotic settings, such IBDs, where proper fermentation reactions may not occur. There are many different subtypes of dietary fibers with large differences in their fermentation profiles. Among them, those belonging to the group of insoluble fibers seem to cause major problems and symptoms in a dysbiotic intestine [16]. Similarly to that which we observed for fiber also happened for milk and dairy products. Lactose can be poorly digested and cause intestinal problems in the presence of a genetic intolerance to lactose or in a dysbiotic gut. The choice to decrease the consumption of lactose, even without medical indications, has been observed in other cohorts of IBD patients and represents an active and conscious choice of these patients in an attempt to reduce their intestinal symptoms after meals [17]. In addition to lactose, other components of dairy products, such as casein proteins, can shift the gut microbiome toward an inflammatory direction [18]. It is much more difficult to explain the reduction of fish consumption observed in the CD group since fish is believed to have predominantly anti-inflammatory effects and its consumption is considered suggested for patients with IBDs [3,19]. Despite this, it is true that unfortunately, in Italian clinical practice, IBD patients are often not followed up by a nutritionist. Much easier to justify is the increased consumption of red meat, already described in IBD patients [17]. The higher presence of red meat could be indirectly linked to the reduction in plant food consumption, in the absence of a correct perception of red meat risks in terms of its negative effects on intestinal health (red and preserved meats in particular).

In our cohort of CD patients, diet was characterized by a decreased presence of fibers and vegetables. This inevitably leads to a significant reduction in the intake of important antioxidant vitamins, such as vit. A (RAE and β-carotene), vit. C and vit. E. These data were confirmed by the decreased ORAC values observed in the whole diet of our CD cohort. The decreased intake of some of these antioxidants, such as vitamin C, has been associated with increased phlogosis indexes in CD patients [20]. Lipophilic vitamins E and A have been shown to protect the intestinal barrier function and ameliorate gut dysbiosis in colitis mice models [21,22]. The intake of vitamins of the B group (in particular B6 and B9) resulted significantly decreased in CD patients, and this can be linked to the decreased presence of plant-based foods in their diet. These vitamins are involved in the metabolism of homocysteine, which is associated with the MTHFR gene, whose polymorphisms have been associated with some IBD populations [23]. The reduced intake of minerals (calcium, potassium, phosphorus, magnesium) that we have highlighted can have various negative effects on IBD patients, such as the increase in the PRAL values of their diet or the increased calciuria. The increased excretion of calcium in the urine, over time, may worsen osteoporosis which, in women with IBDs, appears to be a more serious condition [24]. The decreased iron intake seems to be related to the non-heme form, present in plants, given the lower consumption of vegetables observed in CD patients.

Many different studies have shown that short-chain fatty acids (SCFAs) and long-chain fatty acids (LCFAs) play a vital role in the pathophysiology of CD by various mechanisms including pro- and anti-inflammatory mediators synthesis, intestinal barrier homeostasis and regulation of gene expression [25]. In our CD group, we observed a decreased intake of SCFAs and medium-chain fatty acids (MCFAs). Even if SCFAs are considered protective for CD patients, the majority of absorbed SCFAs derive from bacterial anaerobic fermentations of non-digestible dietary fibers [25]; thus, it is difficult to understand whether the decrease in dietary SCFAs intake could have effects on the pathophysiology of CD. MCFAs have been associated with anti-microbial and anti-inflammatory functions [26]. Partial or complete replacement of dietary LCFAs by MCFAs has been shown to decrease the incidence of spontaneous colitis [25]. Thus, the lower levels of lauric acid (12:0) that we observed in the diet of active CD patients could be considered negative and suggest possible supplementation strategies. Myristic acid (14:0) is a saturated LCFA that could exert desirable anti-inflammatory and protective effects for gut health. Its lower intake in our CD group suggests a possible dietary intervention to restore its ingestion levels [26]. Both our study groups showed a very low ω3/ω6 ratio, and for polyunsaturated fatty acids (PUFAs) concerns, we observed a significant decrease in DHA intake in the CD group. DHA exerts potent anti-inflammatory effects through inhibition of TNFα receptor and TLR4 inflammatory signaling pathways and also through anti-inflammatory lipid mediators synthesis [27]; therefore, it is important that these patients increase their daily DHA intake.

We are aware that in our study we cannot determine how the observed dietary pattern contributed to CD susceptibility. Our study rather aimed to understand if and how the presence of an active CD could influence the nutritional pattern of patients. Overall, our results demonstrated that the decreased nutritional intakes observed in our CD cohort are only partially manifested in the blood, where we only find reduced circulating amounts of iron, potassium, and total cholesterol. The cholesterol datum reveals the presence of an altered intestinal absorption mechanism. In fact, cholesterol intake was slightly higher (without significant differences) in the CD group, while in the blood we find lower circulating amounts. The same explanation could be given to the reduced amino acid blood concentrations, which do not correlate with amino acid intake levels which showed no differences between healthy controls and CD patients. The decreased amino acid absorption can be one of the causes of sarcopenia, often present in active CD patients [28]. We underline that all essential amino acids were found to have reduced plasma concentrations with respect to healthy controls. Taurine was evaluated in the blood of the CD patient group since circulating taurine is known to result also from its increased microbial metabolism by Enterobacteriaceae. The imbalance of this family of bacteria has been commonly observed in the gut microbiota of IBD patients [29]. Our results stress the importance of specific amino acid supplementation, necessary in IBD patients with active disease. For example, the deficiency of arginine has been linked to gut pathophysiology in IBD patients [30]. The results of this study also suggest the need for nutrient and micronutrient supplementation in CD patients: vitamins (A, C, E, B6 and folates), minerals (iron, potassium, calcium, phosphorus, magnesium, copper and iodine) and also fatty acids (especially short- and medium-chain fatty acids and also DHA). This supplementation is necessary to restore intake values at levels similar to those observed in healthy subjects and is crucial to counter blood deficiencies. Even if the adoption of more balanced diets could be effective for IBD patients to avoid some nutritional deficiencies, supplementation is necessary to avoid possible malnutrition status dependent on intestinal malabsorption, a condition that characterizes active Crohn’s patients.

## 4. Materials and Methods

### 4.1. Study Population

Fifty-four adult patients (European, male/female, 18–65 years old) with moderate–severe active Crohn’s disease and with an indication for anti-TNF therapy according to the normal clinical practice and thirty no-IBD controls (European, male/female, 18–65 years old) with no GI disorders, as defined by medical history and standard clinical biochemistry values, afferent to the out-patient clinic. The demographic characteristics of the groups are reported in Table 4.

Disease localization and its severity in enrolled CD patients is shown in Table 5. The study was conducted in conformity with the principles of the Declaration of Helsinki and Good Clinical Practice. The sites involved in enrolment and data collection were the Regional Reference Center for Inflammatory Bowel Diseases, Department of Medical and Surgical Sciences at S. Orsola University Hospital, Bologna, Italy.

### 4.2. Study Design

This study was performed in the context of a one-year prospective observational clinical trial aimed at identifying biomarkers, and predictors of a failure response to commonly used biological therapy in patients with Crohn’s Disease. The full protocol with all study procedures and analysis is available at the site www.clinicaltrial.gov, ClinicalTrials.gov Identifier: NCT02580864. The objectives of this study are purely nutritional and have nothing to do with the patient’s drug therapies or treatment failures. The main objective of this study was to understand if and how the presence of gastrointestinal symptoms modifies patients’ eating habits and if and how these different habits may impact the nutritional deficiencies often highlighted in CD patients. The study was performed at the Regional Reference Center for IBD of the IRCCS Policlinico Sant’Orsola of Bologna, Italy, and was approved by the Regional Ethics Committee (CER): Study code 16/2015/U/Tess approved on 10 February 2015. 

#### 4.2.1. Inclusion Criteria

For IBD patients: informed consent signed, aged between 18 and 65 years old, diagnosis of CD dated at least 3 months prior to screening (involving small intestine and/or colon), confirmed by standard criteria (clinical, endoscopy, laboratory, histology and/or radiology), European ethnicity, indication for anti-TNF therapy, history of corticosteroids dependence or refractoriness and/or immunosuppressants intolerance or failure. For no-IBD patients: informed consent signed, age between 18 and 65 years old, European ethnicity, no medical history of GI disorders, no concomitant treatment with proton pump inhibitors (PPI) or use of antibiotics during the 2 weeks before the enrolment.

#### 4.2.2. Exclusion Criteria

For IBD patients: pregnancy or breast-feeding (on the date of the visit), participation in any CD-related clinical trial at the time of enrolment or during the 3 months before the retrospective observational period, enterocutaneous, abdominal or pelvic active fistulae with abscesses or fistulae likely to require surgery during the study period, bowel surgery, other than appendectomy, within 12 weeks prior to randomization and/or surgery planned or deemed likely for CD patients during the study period, history of extensive colonic resection, and subtotal or total colectomy. Presence of ileostomies, colostomies or rectal pouches, history of more than 3 small bowel resections or diagnosis of short bowel syndrome, known clinically significant stenoses, history or evidence of adenomatous colonic polyps that have not been removed, history or evidence of colonic mucosal dysplasia, chronic use of narcotics for chronic pain, defined as daily use of one or more doses of narcotic containing medication, subjects who had received biologic therapy including adalimumab less than 4 weeks and infliximab less than 8 weeks before baseline, antibiotic treatment within 2 weeks prior to baseline, tube or enteral feeding, elemental diet, or parenteral nutrition within 4 weeks prior to baseline were also considered exclusion criteria. For no-IBD patients: medical history of digestive diseases, medical history of uncontrolled major diseases, digestive (intestinal, gastric, hepatic or pancreatic), renal or metabolic disease, as determined by the medical (screening) visit and a blood chemistry analysis (glucose, total cholesterol, LDL, HDL, triglycerides, ASAT, ALAT, gamma GT, CRP, creatinine), acute illness (e.g., fever, cold, flu) and regular use of medical treatment (pain-killers accepted).

### 4.3. Study Procedures

After a screening visit, IBD patients were followed for one year with 5 trimestral visits; no-IBD patients underwent a single visit. The study procedures are briefly summarized. During each visit, patients consigned a food frequency questionnaire (Supplementary Material File S1) filled out during the seven days before the scheduled visit. To check the accuracy of the information provided, questionnaires were revised with the patient by a nutritionist during each scheduled visit. No nutritional intervention was performed on patients. Moreover, at each visit, blood analysis on fasting patients was performed to detect plasma cholesterol, vitamin B12, vitamin B9 (folic acid), minerals (K, Mg, Fe) and amino acids. 

### 4.4. Food Questionnaires Analysis

Records were entered into a specific nutritional software (Metadieta, METEDA srl, Rome, Italy) to perform bromatological analysis. This software provides estimates of macro- and micronutrient contents based on the Italian food composition database described by Sette et al. [31]. Data obtained were thus exported in an Excel database. For each patient or healthy subject, a 7-day median of each nutrient intake was calculated. Nutrients considered for bromatological analysis were proteins (total, animal, and vegetable), amino acids, saturated and unsaturated fatty acids, carbohydrates (available carbohydrates, amid, oligosaccharides), cholesterol, total fibers, soluble and insoluble fibers, minerals (calcium, iron, sodium, potassium, chloride, chromium, iodine, fluorine, magnesium, copper, selenium), vitamins (A, B1, B2, B6, B9, B12, C, D, E) and total polyphenols. Total daily calories were also calculated. Furthermore, through Metadieta software analysis, we were able to obtain data about the ORAC (Oxygen Radical Absorbance Capacity) and PRAL (Potential Renal Acid Load) of the diets. ORAC is an index that estimates the in vitro antioxidant food activities. PRAL is an indirect measure of the biochemical balance of acidifying and alkalizing molecules contained in foods. A positive PRAL indicates the presence of acidifying potential renal load (typically in meat and cheese), while a negative PRAL is obtained by a large consumption of alkalinizing foods such as vegetables and fruits.

### 4.5. Statistical Analysis

Continuous variables are expressed as mean ± standard deviation (SD) or median and interquartile range (IQR), accordingly. The Kolmogorov–Smirnov assessed the normality of distributions. The Mann–Whitney test was performed to compare medians of 7-day nutrient intakes among patients and healthy subjects. Comparisons of other means or medians were made with Student’s *t*-test or Mann–Whitney test, when appropriate. *p*-values of <0.05 and <0.01 were considered statistically significant. Analyses were performed using IBM SPSS statistic version 28 (IBM SPSS Statistics, Armonk, NY, USA: IBM Corporation).

## 5. Conclusions

Active CD patients tend to adopt unbalanced nutritional patterns, probably caused by an indirect response to their intestinal symptomatology. This unbalanced diet contributes to reducing their intake of some nutrients with antioxidant, anti-dysbiotic and anti-inflammatory effects in the gut. Despite their diet, most of the nutrient deficiencies detected in their blood are dependent on the impairment of their intestinal absorption caused by the disease. Active CD patients need to receive specific nutritional plans to avoid deficiencies and to restore the correct intake of antioxidant, anti-inflammatory and anti-dysbiotic nutrients. These plans, which represent a fundamental therapeutic step for CD patients, should also carefully rebalance the animal/vegetal proteins ratio that resulted as being increased through wrong nutritional choices. Food supplements in CD patients with active disease are required, at least for amino acids and iron supplementation, since their deficiencies appear to be due to intestinal malabsorption and therefore are difficult to be corrected with diet modifications. It would be interesting to compare the diets of patients with active CD with that of CD patients in long-term remission, to verify how the presence of symptoms impacts their food choices.

## Figures and Tables

**Table 1:** Dietary intake in Crohn’s disease (CD) and healthy subjects (HS).

	CD (g/Day) Median (IQR) *n* = 54	HS (g/Day) Median (IQR) *n* = 30	*p*
Soluble fibers	2.3 (1.5–2.9)	2.65 (2.2–3.7)	0.016 *
Insoluble fibers	3.8 (2.7–5.9)	7.3 (4.4–9.6)	<0.0001 ^#^
Dairy products	47.9 (20.0–80.0)	86.4 (48.6–114.3)	0.0046 ^#^
Fish	11.4 (0–41.43)	28.6 (11.4–71.4)	0.0116 *
White meat	24.3 (0.0–48.6)	21.4 (0.0–34.3)	0.5228
Red meat	58.6 (22.9–87.1)	37.9 (11.4–62.9)	0.0494 *
Processed meat	41.4 (20.0–61.4)	31.4 (11.4–52.9)	0.3624
Eggs	0.0 (0.0–17.1)	8.6 (0.0–17.1)	0.2219
Starchy foods (with gluten)	252.9 (167.1–301.4)	245.7 (181.4–306.4)	0.7187
Fruit	74.3 (28.6–128.6)	85.7 (50.0–194.3)	0.3351
Vegetables	65.7 (28.6–152.1)	236.4 (164.3–298.6)	<0.0001 ^#^

Statistically significant at * = *p* < 0.05 or ^#^ = *p* < 0.01.

**Table 2:** Levels of intake for the main dietary lipids.

INTAKE	CD (g/Day) Median (IQR)	HS (g/Day) Median (IQR)	*p*
Cholesterol	0.249 (0.145–0.319)	0.245 (0.176–0.317)	0.7088
Saturated	20.11 (15.06–28.55)	25.18 (16.35–28.88)	0.3677
Unsaturated	31.38 (22.86–43.16)	33.06 (28.93–42.92)	0.3045
Polyunsaturated	7.50 (5.57–9.90)	8.94 (7.00–11.54)	0.0833
Monounsaturated	24.05 (17.08–33.98)	25.41(20.90–32.96)	0.3853
C4:0-C10:0 capric acid	0.22 (0.04–0.41)	0.45 (0.17–0.74)	0.0079 ^#^
C12:0 lauric acid	0.09 (0.02–0.20)	0.19 (0.08–0.33)	0.0183 *
C14:0 myristic acid	0.42 (0.22–0.77)	0.70 (0.37–1.15)	0.0135 *
C16:0 palmitic acid	3.58 (2.36–5.25)	4.81 (3.37–6.65)	0.0505
C18:0 stearic acid	1.60 (0.91–2.56)	2.15 (1.33–3.03)	0.0833
C20:0 arachidic acid	0.06 (0.03–0.13)	0.10 (0.06–0.14)	0.0594
C14:1 myristoleic acid	0.02 (0.01–0.05)	0.03 (0.01–0.06)	0.6033
C16:1 palmitoleic acid	0.36 (0.21–0.60)	0.42 (0.30–0.63)	0.2431
C18:1 oleic acid	12.79 (7.70–17.75)	14.13 (11.18–17.57)	0.1628
C20:1 eicosanoic acid	0.12 (0.08–0.18)	0.13 (0.08–0.24)	0.3695
C22:1 erucic acid	0.01 (0.00–0.02)	0.02 (0.00–0.05)	0.0795
C18:2 linoleic acid	3.50 (2.25–5.26)	3.85 (2.58–5.43)	0.1773
C18:3 α-linolenic acid	0.35 (0.23–0.57)	0.41 (0.33–0.66)	0.1292
C20:4 arachidonic acid	0.13 (0.07–0.20)	0.13 (0.07–0.22)	0.8554
C20:5 EPA	0.01 (0.00–0.03)	0.03 (0.00–0.12)	0.3220
C22:6 DHA	0.01 (0.00–0.02)	0.03 (0.00–0.11)	0.0176 *
Total ω3	0.37 (0.23–0.62)	0.47 (0–0.89)	0.0743
Total ω6	3.63 (2.32–5.46)	3.98 (2.65–5.65)	0.1197
ω3/ω6	0.10 (0.04–0.27)	0.12 (0.02–0.34)	0.09541

Statistically significant at * = *p* < 0.05 or ^#^ = *p* < 0.01; EPA, Eicosapentaenoic acid; DHA Docosahexaenoic acid.

**Table 3:** Blood levels of amino acids compared with intake levels.

	BLOOD LEVELS (mmol/L)			INTAKE (mg/Day)		
	CD Median (IQR)	HS Median (IQR)	*p*	CD Median (IQR)	HS Median (IQR)	*p*
TAU	78.0 (62.0–96.0)	93.0 (82.0–101.0)	<0.001 ^#^			
ASP	3.0 (3.0–4.0)	2.8 (2.0–3.0)	0.0337 *	3456.8 (2336.6–4453.1)	3535.7 (2794.3–4302.2)	0.4897
THR	109.0 (89.0–140.0)	143.0 (113.0–163.0)	0.0012 ^#^	1689.1 (1157.0–2264.6)	1713.2 (1392.1–2090.2)	0.6744
SER	103.0 (86.0–117.0)	115.5 (100.0–133.0)	0.0213 *	2042.1 (1423.4–2770.5)	2116.9 (1633.5–2723.7)	0.5882
GLU	651.0 (583.0–768.0)	775.0 (690.0–887.0)	0.0015 ^#^	8681.8 (6533.3–12266.6)	9189.1 (6967.8–11936.8)	0.6208
PRO	191.5 (153.0–247.0)	222.5 (166.0–263.0)	0.1685	3006.2 (2222.5–4155.8)	3402.6 (2232.4–4093.2)	0.5256
GLY	232.5 (191.0–275.0)	227.5 (195.0–298.0)	0.5316	1797.2 (1191.7–2362.2)	1706.8 (1355.8–2156.4)	0.896
ALA	301.5 (243.0–367.0)	378.5 (300.0–417.0)	0.0126 *	2098.4 (1363.9–2792.9)	1951.1 (1643.8–2492.0)	0.9256
VAL	198.0 (168.0–219.0)	229.0 (197.0–305.0)	<0.001 ^#^	2335.1 (1535.9–2970.9)	2435.7 (1954.5–3010.2)	0.5015
CYS	38.0 (34.0–48.0)	48.5 (41.0–56.0)	0.0017 ^#^	639.6 (459.6–903.8)	662.7 (523.0–853.4)	0.5627
MET	21.0 (17.0–25.0)	25.0 (21.0–29.0)	0.0016 ^#^	1049.8 (707.8–1335.6)	1116.8 (829.0–1324.6)	0.6208
ILE	61.5 (50.0–72.0)	65.0 (51.0–78.0)	0.2507	2037.9 (1323.9–2599.1)	2044.8 (1633.0–2525.2)	0.695
LEU	129.5 (106.0–149.0)	147.0 (111.0–175.0)	0.0483 *	3496.6 (2385.5–4690.3)	3687.2 (2720.9–4568.7)	0.6608
TYR	58.5 (48.0–73.0)	64.0 (58.0–72.0)	0.0866	1878.7 (1065.1–3199.5)	2030.5 (1411.7–2334.0)	0.5754
PHE	57.0 (48.0–67.0)	59.5 (53.0–66.0)	0.3674	1958.7 (1380.3–2679.3)	2178.3 (1586.4–2657.3)	0.4608
ORN	90.5 (69.0–108.0)	81.0 (65.0–104.0)	0.3153			
HIS	69.5 (57.0–80.0)	81.0 (73.0–87.0)	<0.001 ^#^	1327.8 (897.9–1752.3)	1389.2 (1045.5–1689.7)	0.6881
ARG	41.5 (33.0–62.0)	61.5 (45.0–72.0)	0.0072 ^#^	2295.8 (1521.2–3061.8)	2265.4 (1792.8–2786.5)	0.758
LYS	173.5 (140.0–195.0)	181.0 (161.0–201.0)	0.1975	2843.0 (1875.8–3658.9)	2905.2 (2394.0–3674.6)	0.634
CIT	31.0 (25.0–37.0)	37.0 (31.0–48.0)	0.0057 ^#^			

Statistically significant at * = *p* < 0.05 or ^#^ = *p* < 0.01. TAU: Taurine, ASP: Aspartate, THR: Threonine, SER: Serine, GLU: Glutamic acid, PRO: Proline, GLY: Glycine, ALA: Alanine, VAL: Valine, CYS: Cysteine, MET: Methionine, ILE: Isoleucine, LEU: Leucine, TYR: Tyrosine, PHE: Phenylalanine, ORN: Ornithine, HIS: Histidine, ARG: Arginine, LYS: Lysine, CIT: Citrulline.

**Table 4:** Demographics in study cohorts of Crohn’s disease (CD) and healthy subjects (HS).

	CD N (%) or Median (IQR) *n* = 54	HS N (%) or Median (IQR) *n* = 30
Gender:		
Female	35.2%	40.0%
Male	64.8%	60.0%
Age:		
Female	38.8 (20.0–58.0)	35.1 (19.0–52.0)
Male	35.3 (20.0–58.0)	42.5 (19.0–52.0)
BMI:		
Female	22.3 (18.0–26.0)	21.6 (18.0–27.0)
Male	22.8 (18.0–26.0)	23.8 (18.0–27.0)

No statistical differences were observed between CD and HS groups for age or body mass index (BMI) distributions (*p* > 0.05).

**Table 5:** Disease localization and disease activity evaluated by using the Endoscopic Score for Crohn’s Disease (SES-CD).

CD’s Disease Localization	SES-CD Score			
	0–2	3–6	7–15	>15
Ileum	0	2	0	0
Ileocolonic	0	10	22	8
Colon	0	0	11	1

SES-CD Score = 0–2 remission; SES-CD Score = 3–6 mild; SES-CD Score = 7–15 moderate; SES-CD Score > 15 severe disease.

## Data Availability

Raw data are not made available due to ethical restrictions (patient confidentiality).

## Supplementary Materials

The supporting information can be downloaded at: https://www.mdpi.com/article/10.3390/ijms24021494/s1.
