# Supplementary material for: Dietary Habits and Nutrient Deficiencies in a Cohort of European Crohn’s Disease Adult Patients
Source: Int J Mol Sci. 2023 Jan 12;24(2):1494. doi: 10.3390/ijms24021494 (PMC9865585; doi:10.3390/ijms24021494)
Supplement: Supplementary file 1 [file ijms-24-01494-s001.zip › ijms-2102006-supplementary File S1.pdf]

Name & surname: ________________________________ **ID subject:** _______________

Date: _____________________________________ **Day of the week:** _________________

DAY 1

Before breakfast:

TIMES	PLACE	DESCRIPTION OF BEVERAGES / FOODS AND TYPE OF PREPARATION	QUANTITY

Breakfast:

TIMES	PLACE	DESCRIPTION OF BEVERAGES / FOODS AND TYPE OF PREPARATION	QUANTITY

Name & surname: ________________________________ ID subject: _______________

Date: _____________________________________ Day of the week: _________________

DAY 1

Morning Snack:

TIMES	PLACE	DESCRIPTION OF BEVERAGES / FOODS AND TYPE OF PREPARATION	QUANTITY

Lunch:

TIMES	PLACE	DESCRIPTION OF BEVERAGES / FOODS AND TYPE OF PREPARATION	QUANTITY

Name & surname: ________________________________ **ID subject:** _______________

Date: _____________________________________ **Day of the week:** _________________

DAY 1

Afternoon snack:

TIMES	PLACE	DESCRIPTION OF BEVERAGES / FOODS AND TYPE OF PREPARATION	QUANTITY

Dinner:

TIMES	PLACE	DESCRIPTION OF BEVERAGES / FOODS AND TYPE OF PREPARATION	QUANTITY

Name & surname: ___________________________________ ID subject: _________________

Date: ____ Day of the week: ___________________

DAY 1

After dinner:

TIMES	PLACE	DESCRIPTION OF BEVERAGES / FOODS AND TYPE OF PREPARATION	QUANTITY

During the night:

TIMES	PLACE	DESCRIPTION OF BEVERAGES / FOODS AND TYPE OF PREPARATION	QUANTITY

Comments (e.g. recipes)

____
____
____
____
____
____

Name & surname: ________________________________ **ID subject:** _______________

Date: _____________________________________ **Day of the week:** _________________

DAY 2

Before breakfast:

TIMES	PLACE	DESCRIPTION OF BEVERAGES / FOODS AND TYPE OF PREPARATION	QUANTITY

Breakfast:

TIMES	PLACE	DESCRIPTION OF BEVERAGES / FOODS AND TYPE OF PREPARATION	QUANTITY

Name & surname: ________________________________ ID subject: _______________

Date: _____________________________________ Day of the week: _________________

DAY 2

Morning Snack:

TIMES	PLACE	DESCRIPTION OF BEVERAGES / FOODS AND TYPE OF PREPARATION	QUANTITY

Lunch:

TIMES	PLACE	DESCRIPTION OF BEVERAGES / FOODS AND TYPE OF PREPARATION	QUANTITY

Name & surname: _________________________________ **ID subject:** ________________

Date: ______________________________________ **Day of the week:** __________________

DAY 2

Afternoon snack:

TIMES	PLACE	DESCRIPTION OF BEVERAGES / FOODS AND TYPE OF PREPARATION	QUANTITY

Dinner:

TIMES	PLACE	DESCRIPTION OF BEVERAGES / FOODS AND TYPE OF PREPARATION	QUANTITY

Name & surname: ________________________________ ID subject: _______________

Date: _____________________________________ Day of the week: _________________

DAY 2

After dinner:

TIMES	PLACE	DESCRIPTION OF BEVERAGES / FOODS AND TYPE OF PREPARATION	QUANTITY

During the night:

TIMES	PLACE	DESCRIPTION OF BEVERAGES / FOODS AND TYPE OF PREPARATION	QUANTITY

Comments (e.g. recipes)

____

____

____

____

____

Name & surname: ___________________________________ **ID subject: _________________**

Date: ____ **Day of the week: ___________________**

DAY 3

Before breakfast:

TIMES	PLACE	DESCRIPTION OF BEVERAGES / FOODS AND TYPE OF PREPARATION	QUANTITY

Breakfast:

TIMES	PLACE	DESCRIPTION OF BEVERAGES / FOODS AND TYPE OF PREPARATION	QUANTITY

Name & surname: ___________________________________ **ID subject: _________________**

Date: ____ **Day of the week: ___________________**

DAY 3

Morning Snack:

TIMES	PLACE	DESCRIPTION OF BEVERAGES / FOODS AND TYPE OF PREPARATION	QUANTITY

Lunch:

TIMES	PLACE	DESCRIPTION OF BEVERAGES / FOODS AND TYPE OF PREPARATION	QUANTITY

Name & surname: ________________________________ ID subject: _______________

Date: _____________________________________ Day of the week: _________________

DAY 3

Afternoon snack:

TIMES	PLACE	DESCRIPTION OF BEVERAGES / FOODS AND TYPE OF PREPARATION	QUANTITY

Dinner:

TIMES	PLACE	DESCRIPTION OF BEVERAGES / FOODS AND TYPE OF PREPARATION	QUANTITY

Name & surname: ___________________________________ ID subject: _________________

Date: ____ Day of the week: ___________________

DAY 3

After dinner:

TIMES	PLACE	DESCRIPTION OF BEVERAGES / FOODS AND TYPE OF PREPARATION	QUANTITY

During the night:

TIMES	PLACE	DESCRIPTION OF BEVERAGES / FOODS AND TYPE OF PREPARATION	QUANTITY

Comments (e.g. recipes)

____
____
____
____
____
____

Name & surname: __________________________________ **ID subject: ________________**

Date: _______________________________________ **Day of the week: __________________**

DAY 4

Before breakfast:

TIMES	PLACE	DESCRIPTION OF BEVERAGES / FOODS AND TYPE OF PREPARATION	QUANTITY

Breakfast:

TIMES	PLACE	DESCRIPTION OF BEVERAGES / FOODS AND TYPE OF PREPARATION	QUANTITY

Name & surname: ________________________________ ID subject: _______________

Date: _____________________________________ Day of the week: _________________

DAY 4

Morning Snack:

TIMES	PLACE	DESCRIPTION OF BEVERAGES / FOODS AND TYPE OF PREPARATION	QUANTITY

Lunch:

TIMES	PLACE	DESCRIPTION OF BEVERAGES / FOODS AND TYPE OF PREPARATION	QUANTITY

Name & surname: ________________________________ **ID subject:** _______________

Date: _____________________________________ **Day of the week:** _________________

DAY 4

Afternoon snack:

TIMES	PLACE	DESCRIPTION OF BEVERAGES / FOODS AND TYPE OF PREPARATION	QUANTITY

Dinner:

TIMES	PLACE	DESCRIPTION OF BEVERAGES / FOODS AND TYPE OF PREPARATION	QUANTITY

Name & surname: ________________________________ **ID subject:** _______________

Date: _____________________________________ **Day of the week:** _________________

DAY 4

After dinner:

TIMES	PLACE	DESCRIPTION OF BEVERAGES / FOODS AND TYPE OF PREPARATION	QUANTITY

During the night:

TIMES	PLACE	DESCRIPTION OF BEVERAGES / FOODS AND TYPE OF PREPARATION	QUANTITY

Comments (e.g. recipes)

____
____
____
____
____
____

Name & surname: ________________________________ **ID subject: _______________**

Date: _____________________________________ **Day of the week: _________________**

DAY 5

Before breakfast:

TIMES	PLACE	DESCRIPTION OF BEVERAGES / FOODS AND TYPE OF PREPARATION	QUANTITY

Breakfast:

TIMES	PLACE	DESCRIPTION OF BEVERAGES / FOODS AND TYPE OF PREPARATION	QUANTITY

Name & surname: ____________________________________ ID subject: _________________

Date: ____ Day of the week: ___________________

DAY 5

Morning Snack:

TIMES	PLACE	DESCRIPTION OF BEVERAGES / FOODS AND TYPE OF PREPARATION	QUANTITY

Lunch:

TIMES	PLACE	DESCRIPTION OF BEVERAGES / FOODS AND TYPE OF PREPARATION	QUANTITY

Name & surname: ________________________________ **ID subject:** _______________

Date: _____________________________________ **Day of the week:** _________________

DAY 5

Afternoon snack:

TIMES	PLACE	DESCRIPTION OF BEVERAGES / FOODS AND TYPE OF PREPARATION	QUANTITY

Dinner:

TIMES	PLACE	DESCRIPTION OF BEVERAGES / FOODS AND TYPE OF PREPARATION	QUANTITY

Name & surname: ________________________________ ID subject: _______________

Date: _____________________________________ Day of the week: _________________

DAY 5

After dinner:

TIMES	PLACE	DESCRIPTION OF BEVERAGES / FOODS AND TYPE OF PREPARATION	QUANTITY

During the night:

TIMES	PLACE	DESCRIPTION OF BEVERAGES / FOODS AND TYPE OF PREPARATION	QUANTITY

Comments (e.g. recipes)

____

____

____

____

____

Name & surname: ________________________________ ID subject: _______________

Date: _____________________________________ Day of the week: _________________

DAY 6

Before breakfast:

TIMES	PLACE	DESCRIPTION OF BEVERAGES / FOODS AND TYPE OF PREPARATION	QUANTITY

Breakfast:

TIMES	PLACE	DESCRIPTION OF BEVERAGES / FOODS AND TYPE OF PREPARATION	QUANTITY

Name & surname: ________________________________ ID subject: _______________

Date: _____________________________________ Day of the week: _________________

DAY 6

Morning Snack:

TIMES	PLACE	DESCRIPTION OF BEVERAGES / FOODS AND TYPE OF PREPARATION	QUANTITY

Lunch:

TIMES	PLACE	DESCRIPTION OF BEVERAGES / FOODS AND TYPE OF PREPARATION	QUANTITY

Name & surname: ________________________________ ID subject: _______________

Date: _____________________________________ Day of the week: _________________

DAY 6

Afternoon snack:

TIMES	PLACE	DESCRIPTION OF BEVERAGES / FOODS AND TYPE OF PREPARATION	QUANTITY

Dinner:

TIMES	PLACE	DESCRIPTION OF BEVERAGES / FOODS AND TYPE OF PREPARATION	QUANTITY

Name & surname: ________________________________ **ID subject:** _______________

Date: _____________________________________ **Day of the week:** _________________

DAY 6

After dinner:

TIMES	PLACE	DESCRIPTION OF BEVERAGES / FOODS AND TYPE OF PREPARATION	QUANTITY

During the night:

TIMES	PLACE	DESCRIPTION OF BEVERAGES / FOODS AND TYPE OF PREPARATION	QUANTITY

Comments (e.g. recipes)

____
____
____
____
____
____

Name & surname: ________________________________ **ID subject:** ________________

Date: _____________________________________ **Day of the week:** __________________

DAY 7

Before breakfast:

**TIMES**	**PLACE**	**DESCRIPTION OF BEVERAGES / FOODS AND TYPE OF PREPARATION**	**QUANTITY**

Breakfast:

**TIMES**	**PLACE**	**DESCRIPTION OF BEVERAGES / FOODS AND TYPE OF PREPARATION**	**QUANTITY**

Name & surname: ________________________________ ID subject: _______________

Date: _____________________________________ Day of the week: _________________

DAY 7

Morning Snack:

**TIMES**	**PLACE**	**DESCRIPTION OF BEVERAGES / FOODS AND TYPE OF PREPARATION**	**QUANTITY**

Lunch:

**TIMES**	**PLACE**	**DESCRIPTION OF BEVERAGES / FOODS AND TYPE OF PREPARATION**	**QUANTITY**

Name & surname: ___________________________________ **ID subject:** _________________

Date: ____ **Day of the week:** ___________________

DAY 7

Afternoon snack:

TIMES	PLACE	DESCRIPTION OF BEVERAGES / FOODS AND TYPE OF PREPARATION	QUANTITY

Dinner:

TIMES	PLACE	DESCRIPTION OF BEVERAGES / FOODS AND TYPE OF PREPARATION	QUANTITY

Name & surname: ________________________________ **ID subject:** _______________

Date: _____________________________________ **Day of the week:** _________________

DAY 7

After dinner:

TIMES	PLACE	DESCRIPTION OF BEVERAGES / FOODS AND TYPE OF PREPARATION	QUANTITY

During the night:

TIMES	PLACE	DESCRIPTION OF BEVERAGES / FOODS AND TYPE OF PREPARATION	QUANTITY

Comments (e.g. recipes)

____

____

____

____

____
